# Evaluating the validity of multiple imputation for missing physiological data in the national trauma data bank

**DOI:** 10.4103/0974-2700.44774

**Published:** 2009

**Authors:** Lynne Moore, James A Hanley, André Lavoie, Alexis Turgeon

**Affiliations:** 1 Department of Epidemiology and Biostatistics. McGill University, Montreal, Quebec, Canada; 2 Unité de Traumatologie-Urgence-Soins Intensifs, Centre de recherche du CHA (Hôpital de l'Enfant-Jésus), Université Laval, Quebec City, Quebec, Canada; 3 Département d'Anesthésie, Division de Soins Intensifs, Université Laval, Quebec City, Quebec, Canada

**Keywords:** Missing data, multiple imputation, risk adjustment, trauma registry

## Abstract

**Background:**

The National Trauma Data Bank (NTDB) is plagued by the problem of missing physiological data. The Glasgow Coma Scale score, Respiratory Rate and Systolic Blood Pressure are an essential part of risk adjustment strategies for trauma system evaluation and clinical research. Missing data on these variables may compromise the feasibility and the validity of trauma group comparisons.

**Aims:**

To evaluate the validity of Multiple Imputation (MI) for completing missing physiological data in the National Trauma Data Bank (NTDB), by assessing the impact of MI on 1) frequency distributions, 2) associations with mortality, and 3) risk adjustment.

**Methods:**

Analyses were based on 170,956 NTDB observations with complete physiological data (observed data set). Missing physiological data were artificially imposed on this data set and then imputed using MI (MI data set). To assess the impact of MI on risk adjustment, 100 pairs of hospitals were randomly selected with replacement and compared using adjusted Odds Ratios (OR) of mortality. OR generated by the observed data set were then compared to those generated by the MI data set.

**Results:**

Frequency distributions and associations with mortality were preserved following MI. The median absolute difference between adjusted OR of mortality generated by the observed data set and by the MI data set was 3.6% (inter-quartile range: 2.4%-6.1%).

**Conclusions:**

This study suggests that, provided it is implemented with care, MI of missing physiological data in the NTDB leads to valid frequency distributions, preserves associations with mortality, and does not compromise risk adjustment in inter-hospital comparisons of mortality.

## INTRODUCTION

The National Trauma Data Bank (NTDB) is the biggest aggregation of trauma data currently available, now containing over two million observations from 405 US hospitals.[1] It is used to assess trauma systems and institutional performance, and for clinical and epidemiological research, all requiring risk adjustment. The outcome most commonly studied in trauma populations is hospital mortality. The risk of mortality following injury can be described according to physiological reserve, anatomical injury severity, and physiological response to injury.[2] The latter is traditionally quantified by three variables measured on arrival at the emergency department: the Glasgow Coma Scale (GCS) score, Respiratory Rate (RR), and Systolic Blood Pressure (SBP). Unfortunately, like all trauma registries, the NTDB suffers from a high prevalence of missing physiological data.

In the presence of missing physiological data, trauma group comparisons are often performed by excluding observations with missing data or omitting physiological variables from analyses. The former strategy may generate biased effect estimates since patients with missing data differ systematically from those with non-missing data[3] and the latter may result in residual confounding due to incomplete adjustment for baseline risk.[4] A solution to the problem of missing physiological data is to predict missing data from other information available in the patient file. This is known as data imputation and can be performed with several methods, the most interesting of which is Multiple Imputation (MI).[5] MI simulates multiple values for missing data using a prediction model and therefore accounts for the uncertainty surrounding missing data values.[5]

The objective of the present study was to assess the validity of MI for imputing missing GCS, RR and SBP in the NTDB by evaluating the impact of MI on 1) frequency distributions, 2) associations with hospital mortality, and 3) risk adjustment.

## MATERIALS AND METHODS

### Selection and description of participants

The NTDB is an aggregation of data from institutional trauma registries provided on a voluntary basis by over 400 USA hospitals that treat trauma. The NTDB contains information on patient demographics, circumstances of injury, physiological response to injury, anatomic injuries, clinical interventions and discharge destinations. In some NTDB hospitals, anatomic injuries are coded with the Abbreviated Injury Scale (AIS);[6] in others, the International Classification of Diseases (ICD) is used. The study population was based on data collected in 2006 (version 7.0 of the NTDB). Analyses were limited to observations with at least one AIS code or at least one ICD injury code (800-959; excluding late effects, foreign bodies, complications and burns). Children (< 17 years of age) were excluded as their physiological reaction to injury differs from that of adults. Deaths on arrival were excluded as information on their injuries is often incomplete and isolated hip fractures were excluded because they are commonly considered to be a consequence of chronic disease rather than trauma.[7] To standardize heterogeneous hospital inclusion criteria, analysis were also restricted to observations respecting trauma registry inclusion criteria recommended by the American College of Surgeons: hospital stay > 48 h, intensive care unit admission, death, or transfer.[7] The study was approved by our institutional research ethics committee.

### Technical details

The NTDB includes variables for the Motor, Verbal, and Eye components of the GCS, RR, and SBP evaluated in the emergency department. Motor, Verbal, and Eye components of the GCS were considered missing if data were absent, aberrant, or if the patient was sedated at the time of evaluation. In addition, the Verbal component was considered missing if the patient was intubated at the time of evaluation. RR was considered missing if data were absent, aberrant (< 0 or >120) or the patient was intubated and/or sedated at the time of evaluation. SBP was considered missing if data were absent or aberrant (< 0 or >300).

Anatomical injury severity was described with AIS severity scores for patients with AIS injury coding.[6] For hospitals without AIS coding, the ICD Injury Severity Score was used.[8] There are several versions of this score. We used the independent survival proportion of the most severe injury as this version has been reported to describe mortality the most accurately.[9–11] The severity of head injuries was quantified separately from that of injuries to other body regions as the GCS and RR are more likely to be clinically abnormal in patients with head injuries.

For analysis, the study population was restricted to observations with nonmissing emergency department GCS, RR, and SBP values. This data set will be referred to as the observed data set, and all parameters generated by this data set as observed parameters.

### Imposition of missing data

Missing values were artificially imposed in the observed data set on the Motor, Verbal, and Eye components of the GCS as well as on RR and SBP. Missing data were conditioned on the frequency distributions of physiological variables. Information provided by emergency physicians suggested that physiological data are most likely to be missing for patients undergoing pre-hospital intubation/sedation, for patients in a critical condition on arrival at the emergency department, and for patients with minor extra-cranial trauma. We therefore assumed that data were more likely to be missing for severely injured patients (low GCS, low RR, and low SBP), followed by patients with minor injuries (high GCS, 10<RR< 29, and SBP≥90) and less likely to be missing for patients with moderate injuries. Using this information, we randomly imposed missing data on a proportion of observations that varied according to the values of each variable [Table 1]. Proportions were chosen to produce realistic variation and a global proportion equal to that present in the full NTDB. For interval variables (RR and SBP), missing data were imposed according to categories commonly considered to correspond to high–low risk groups.[12]

**Table 1:** Prevalence of imposed missing data according to the frequency distribution of hysiological variables

Variable	Value	% imposed missing data
GCS: Motor	1 – No motor response	35
	2 – Extension to pain	20
	3 – Flexion to pain	10
	4 – Withdrawal from pain	5
	5 – Localising pain	5
	6 – Obeys commands	13

GCS: Verbal	1 – No verbal response	45
	2 – Incomprehensible sounds	30
	3 – Inappropriate words	15
	4 – Confused	15
	5 – Orientated	20

GCS: Eye	1 – No eye opening	45
	2 – Eye opening to pain	30
	3 – Eye opening to verbal command	15
	4 – Eyes open spontaneously	20

RR	0	21
	1–5	11
	6–9	6
	≥30	6
	10–29	11

SBP	0	5
	1–49	4
	50–75	3
	76–89	2
	≥90	3

### MI methodology

Prior to MI implementation, a data imputation model was defined. Analyses were performed with the observed data set (with imposed missing values) to identify variables associated with the GCS, RR, and SBP, and variables associated with the fact that they were missing.[13]

MI was implemented using PROC MI (SAS Institute, Cary, version 9.1). The Markov Chain Monte Carlo method was used with a noninformative prior and a single chain. The multivariate normal model was used for all imputations.[14] Convergence and independence of imputed values were assessed with time series and autocorrelation plots of the mean and variance of physiological variables.[13]

The imputation model included the following variables: Age (<55, 55–64, 65–74, 75–84, ≥85); the maximum AIS for head injuries or, in the absence of AIS coding, the ICD injury severity score for head injuries; the maximum AIS or the ICD injury severity score for injuries to body regions other than the head; transfer status; injury mechanism (motor vehicle collision, fall, gunshot/stabbed, other); total length of stay in days (log transformed, missing for deaths); length of stay in the intensive care unit in days (log transformed, missing for deaths); duration of mechanical ventilator in days (log transformed, missing for deaths); disposition at discharge from the emergency department (died in emergency department, ward, intensive care unit, operating room, other); and discharge disposition (died following admission, home, rehabilitation, skilled nursing facility/nursing home, hospital transfer, other).

Motor, Verbal, and Eye components of the GCS were imputed as a series of indicator variables with 1 (normal level of consciousness) as the reference category. RR was imputed using a Box Cox transformation with lambda = 0.75 to normalize its negatively skewed distribution.

SBP was imputed as a quantitative variable with no transformation as it is approximately Gaussian.

The data set with missing physiological data imputed with MI will be referred to as the MI data set and all parameters generated by the data set as MI parameters.

### Statistical analysis

MI accuracy was assessed by comparing observed results to those generated by MI for the following: i) frequency distributions; ii) associations with mortality; and iii) risk adjustment.

For i), the frequency distributions of RR and SBP were estimated using kernel density estimation with PROC KDE (SAS Institute, Cary, version 9.1). A Sheather-Jones plug-in was used with a bandwidth of 0.4 and 1.8, respectively. For ii), as the GCS has a discrete distribution, percent mortality along with exact binomial confidence intervals were calculated for each GCS value. For RR and SBP, cubic smoothing splines were used to model their non-monotonic relation to mortality. The latter were implemented with a fixed smoothing parameter equivalent to 4 degrees of freedom in logistic generalized additive models[15] using PROC GAM (SAS Institute, Cary, version 9.1).

For iii), we used inter-hospital comparisons of adjusted mortality. Hospital comparisons were used because hospital profiling represents a common use of trauma registry data and leads to important heterogeneity in the risk profiles to be compared. Risk adjustment was based on a logistic generalized additive model including the GCS, RR, SBP, the AIS severity score of the two most severe injuries, the body region of the most severe injury, and age. Quantitative variables were modeled with cubic smoothing splines to accommodate nonlinear relations to the logit of mortality. Risk-adjusted mortality was compared over hospitals using Odds Ratios (OR). We were interested in the difference between observed OR and OR generated by the MI dataset. A difference in OR estimates of over 10% was considered to be important.[16] To obtain effect estimates with acceptable precision, analyses were restricted to hospitals with at least 1000 observations.

Hospital comparisons were performed in the following sequence: 1) Two hospitals were selected at random and compared by adding an indicator variable to the risk adjustment model described above, 2) an adjusted OR and a standard error estimate were generated for the indicator variable, 3) absolute difference in OR and difference in standard error were calculated, 4) Repeat random sampling of hospitals with replacement was used to perform 100 such pairwise comparisons, and 5) The median absolute percent difference in OR estimates and the median percent difference in standard error were calculated over the 100 comparisons.

In addition to MI, we assessed the impact of two alternative strategies for dealing with missing physiological data on risk adjustment: i) excluding observations with missing physiological data (listwise deletion) and ii) omitting physiological variables from the risk adjustment model.

## RESULTS

The full NTDB comprised 104 level I, 105 level II and 28 level III trauma centers and 178 non-designated hospitals for a total of 230,955 observations. There were 12,848 in-hospital deaths (5.6%). The Motor, Verbal, and Eye components of the GCS were missing in 13.0%, 21.4%, and 20.3% of patients, respectively. RR and SBP were missing for 10.8% and 3.2% of patients, respectively.

The prevalence of missing physiological data appeared to increase with the severity of head injuries [Table 2]. Around 50% of emergency department deaths had a missing GCS or RR.

**Table 2:** Distribution of patient characteristics and prevalence of missing glasgow coma scale, respiratory rate, or systolic blood pressure (full data set; *n* = 230,955)

Variable		N (%)	Missing GCS (%)	Missing RR (%)	Missing SBP (%)
Age	<65	183404 (79.4)	22.0	11.0	2.90
	≥65	47551 (20.6)	23.9	10.0	4.37

Maximum AIS for head injuries*	0	39837 (17.3)	38.4	8.89	5.15
	1–3	15665 (24.0)	44.7	9.58	2.30
	4–6	9862 (15.1)	50.4	22.8.	3.89

Maximum* AIS for other body regions	0 – no injury	5384 (2.3)	48.8	15.6	5.87
	1–3	51767 (79.2)	40.9	9.73	4.42
	4–6	8213 (12.6)	42.2	17.2	2.36

Emergency department destination	Death	2998 (1.3)	46.3	50.3	8.57
	Ward	104052 (45.1)	19.6	6.72	3.57
	Intensive care	62400 (27.0)	27.3	15.9	1.87
	Operating room	38573 (16.7)	23.0	11.8	2.09
	Other	19405 (8.4)	15.6	9.60	7.35
	Not coded	3527 (1.5)	31.7	2.61	0.37

Discharge destination	Death	11657 (5.1)	38.5	32.6	4.38
	Home	147761 (64.0)	20.0	7.82	2.81
	Rehabilitation	26165 (11.3)	26.0	15.6	3.72
	Long term care	24884 (10.8)	24.6	8.7	4.27
	Hospital transfer	5776 (2.5)	21.0	14.3	2.60
	Other	6248 (2.7)	25.3	11.7	1.84
	Not coded^†^	8464 (3.7)	26.9	20.5	5.16

GCS: Glasgow Coma Scale score; RR: Respiratory Rate; SBP: Systolic Blood Pressure; AIS, Abbreviated Injury Scale score

* 165,591 patients had no AIS coding; GCS, RR, and SBP were missing for 19.7% of these patients

† Includes 2998 emergency department deaths

Source: Data from reference 6

After exclusion of observations with missing physiological data, 170,956 observations (74%) were available for analyses, including 7,284 deaths (4.3%). Following imposition of missing data, the Motor, Verbal, and Eye components of the GCS had 13.5%, 21.2%, and 21.5% missing data, respectively. The RR and SBP had 11.2% and 3.1% of missing data, respectively.

Autocorrelation and time series plots generated during the MI process indicated rapid convergence to a stable distribution and rapid dissipation of autocorrelation for all variables. Given the fraction of missing information associated with the Motor, Verbal, and Eye components of the GCS, RR, and SBP, five imputes led to relative efficiency estimates of between 92% and 99%.

**Figure 1:**
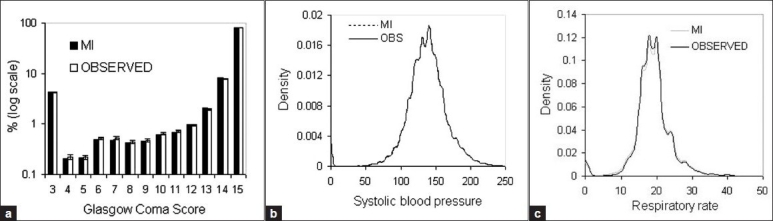
Frequency distributions of physiological variables in observed and multiple imputation (MI) datasets (*n* = 170,956)

MI slightly under-estimated the frequency of GCS values 4 to 11 and overestimated values 12–14 [Figure 1a]. The RR distribution generated by MI had slightly heavier tails than the observed distribution [Figure 1b]. The SBP frequency distribution generated by MI was practically indistinguishable from the observed distribution [Figure 1c].

**Figure 2:**
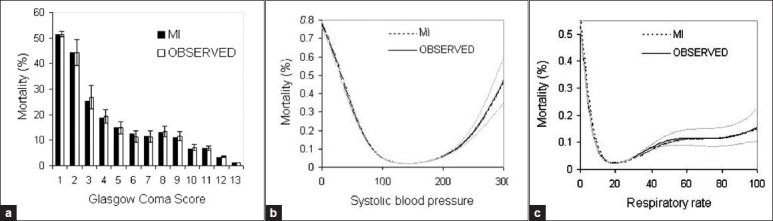
Associations of physiological variables with mortality in observed and multiple imputation (MI) datasets (*n* = 170,956)

MI Mortality proportions were within 95% confidence intervals of observed proportions for the whole range of GCS values [Figure 2a]. Imputed RR led to an underestimation in mortality risk from 30<RR< 60 but remained within 95% confidence intervals for all other values [Figure 2b]. The functional relationship between SBP and mortality risk remained practically unchanged following MI [Figure 2c].

In risk-adjusted inter-hospital comparisons of mortality, MI led to a median absolute difference in OR below 10% [Table 3]. As expected, standard errors of MI parameter estimates were higher than those associated with observed parameter estimates.

**Table 3:** Divergence in the results of risk-adjusted inter-hospital mortality comparisons from the observed data set

		MI	Listwise deletion	Omission of physiological variables
% difference in OR	Median*	3.6	18.8	60.5
	(Q1;Q3)	(2.4;6.1)	(8.2;41)	(27;138)

% difference in SE	Median^†^	−0.08	−66.6	20.6
	(Q1;Q3)	(−0.6;0.6)	(-82;-57)	(12;28)

OR, Odds Ratio; SE, Standard Error; Q1;Q3; Inter-quartile range, MI, Multiple Imputation

* For MI = MEDIAN|(( observed OR − MI OR)/observed OR) × 100) |

† For MI = MEDIAN (((observed SE − MI SE)/observed SE) × 100)

Listwise deletion of observations with missing physiological data and omission of physiological variables from risk adjustment models both led to a median absolute difference in OR over 10% [Table 3].

## DISCUSSION

Despite its limitations,[7] the NTDB is an invaluable source of information that will continue to improve our understanding of the societal burden of injury and facilitate the improvement of patient care.[17] Information on physiological response to injury is an important independent predictor of mortality and other trauma outcomes, such as functional capacity,[18] quality of life[19] and costs.[20] It is therefore an essential part of risk adjustment strategies in clinical and epidemiological research as well as performance evaluation. To date, analyses using the NTDB and any other trauma registry have been impaired by missing physiological data. The results of this study indicate that MI may be a valid solution to the problem.

GCS, RR, and SBP frequency distributions generated by MI were very close to observed distributions, and the relation of these variables to mortality was preserved following MI. Furthermore, the results of pairwise hospital mortality comparisons suggest that MI of physiological variables used for risk adjustment may lead to non-biased trauma group comparisons. In addition, the OR generated by MI had a higher standard error than the observed coefficient, which indicates that MI really does take account of the uncertainty surrounding missing data by inflating resulting variance estimates. Results also indicate that two common strategies for dealing with missing physiological data, deleting observations with missing data and omitting physiological variables from the risk adjustment model, lead to unacceptable bias in OR estimates.

This study adds to accumulating evidence that MI can offer a valid solution to the problem of missing physiological data in trauma registries. Missing pre-hospital GCS, RR, and SBP simulated via MI have been shown to have good agreement with values given on ambulance run sheets[21] and MI of missing physiological data in the National Pediatric Trauma Registry has been shown to improve the accuracy of mortality prediction.[22] In addition, MI of emergency department GCS has been shown to lead to accurate frequency distributions and associations with mortality.[3]

MI offers many advantages. The use of multiple imputes addresses the uncertainty surrounding the missing value; if information in the prediction model is limited, resulting imputations will be heterogeneous, which will result in robust variance estimates in ensuing analyses. MI uses information on any number of auxiliary variables whether they have missing values or not; it simultaneously imputes data for all variables with missing values. Finally, MI generates imputed values that can be added to the trauma registry database and used in future analyses as long as covariance structures are respected.

### Limitations

The validity of MI relies on the postulate that physiological variables are Missing Completely At Random (completely arbitrary, missingness does not depend on the values of Z or on auxiliary variables) or Missing At Random (missingness depends on the values of Z but can be explained by auxiliary variables). MI may therefore not work well if data are Not Missing At Random (missingness depends on the values of Z and cannot be explained by auxiliary variables). Unfortunately, this postulate can never be verified formally. Reasons for missing physiological data in a trauma registry are varied: 1) inability to evaluate the GCS and RR in patients who are intubated and/or sedated prior to arrival at the hospital and patients under the influence of drugs/alcohol, 2) lack of time to perform evaluations in early deaths, and 3) lack of evaluation in patients with minor extracranial trauma. Physiological data can also be missing in a random manner because it was not inscribed in patients' medical file or data collectors missed the information in the file. In addition, the prevalence and the mechanism of missing data vary over ED physicians, data collectors and hospitals. While it can never be formally verified, we hypothesize that the mechanism behind missing physiological data in trauma registries probably lies between Missing At Random and Not Missing At Random as other registry variables will provide some but not all information on missing data.

Evidently, we could not exactly reproduce the missing data mechanism present in the NTDB. However, by conditioning missing data on the frequency distribution of physiological variables we were able to reproduce a clinically plausible Missing At Random data mechanism. While it is possible to impute data that are Not Missing At Random through specification of the missing data mechanism,[23] results are highly dependent upon the parameters used to describe that mechanism.[13] Evidently, the diverse reasons behind missing physiological data, many of which are not quantified in trauma registries, make the data mechanism very difficult to describe. Simulation studies have suggested that MI performs well even when data are Not Missing At Random. [3–13]

MI does have other limitations that should not be ignored. First, imputations are valid for population comparisons and are not designed to be accurate for individuals. Second, the analyst must verify convergence to a stable distribution to ensure success. Third, care must be taken when using imputations in future inference. If the inference model includes variables or interaction terms that were not included in the imputation process but are associated with the outcome under study and with physiological variables, parameter estimates can be biased. The imputation process should always be reviewed therefore, before performing inference with imputed data.

The NTDB is hampered by the threat of selection bias, caused by voluntary participation and heterogeneous inclusion criteria, and by information bias, caused by non-standardized coding practices.[7] Information bias, could pose a threat to the validity of imputed data. In particular, information on two important determinants of missing physiological data, pre-hospital and emergency department intubation/sedation and alcohol/drug intoxication, was incomplete. The fact that such promising results were observed in a database with data quality problems is an excellent demonstration of the robustness of MI and suggests it will perform well in other trauma registries. In addition, we have shown that MI of physiological data can be performed even if AIS injury coding is not available by using ICD survival probabilities to quantify anatomical injury severity.

## CONCLUSIONS

The best solution to missing physiological data in trauma registries remains avoidance. Therefore, efforts should constantly be made to facilitate the collection of complete data. However, it is probably unrealistic to hope that missing data may one day be eradicated from trauma registries. This study adds to accumulating evidence that MI may provide a valid solution to the problem of missing physiological data, provided it is implemented with care.
